# Proceedings of the South Carolina State Dental Association

**Published:** 1870-06

**Authors:** Thos. T. Moorse

**Affiliations:** Corresponding Secretary.


### ARTICLE IV.

### *Proceedings of the South Carolina State Dental Association.*

Held in Columbia on 5th, 6th, and 7th of April, 1870.

BY **THOS**. T. MOORSE, D.D.S., Corresponding Secretary.

A call having been made (by the members of an Association previously formed) for a new Association that would embrace every portion of the State, on the day appointed a large number of Dentists assembled in Columbia, and proceeded to form a "State Dental Association."

The following officers were elected to serve the ensuing year:

*President*, Dr. J. B. Patrick, Charleston; *First Vice President*, Dr. Wm. C. Wardlaw, Abbeville; *Second Vice President*, Dr. H. R. Hanbery, Barnwell; *Corresponding Secretary*, Dr. Thos. T. Moorse, Columbia; *Recording Secretary*, Dr. O. J. Bond, Marion; *Treasurer*, Dr. Tho. F. Chupein, Charleston.

The President appointed the following standing committees:

*Executive Committee.*—Drs. D. L. Boozer, G. F. Wright, A. K. Durham, N. Simms, Sam'l A White.

*Committee on Membership.*—Drs. Wm. L. Reynolds, E. C. Jones, J. R. Thompson, B. C. Hart, Geo. H. Winkler.

*Committee on Operative Dentistry.*—Drs. O. J. Bond, W. C. Wardlaw, Thos. T. Moorse, W. S. Brown, Thos. F. Chupein.

*Committee on Mechanical Dentistry.*—Drs. R. S. Whaley,

W. A. Fallaw, H. R. Hanbery, J. H. Alexander, B. A. Muckenfuss.

The following were elected delegates to the "Southern Dental Association;" Drs. J. B. Patrick and Wm. C. Wardlaw.

Delegates to the "American Dental Association;" Drs. Thos. T. Moorse and H. R. Hanbery.

The deligates to the "Southern" and "American Dental Associations," were instructed to use their influence in having a committee appointed to petiiton Congress to appoint Dentists in the Army and Navy of the U. S.

After a very harmonious meeting, the Association adjourned, to hold its *Semi-annual* meeting in Charleston, in Nov. next, and, its *Annual* meeting in Columbia, in the first Tuesday in May, 1871 at 8 P. M.

---

